# Exposure to Mercury in Workers and the Population Surrounding Gold Mining Areas in the Mojana Region, Colombia

**DOI:** 10.3390/ijerph15112337

**Published:** 2018-10-23

**Authors:** Sonia Mireya Díaz, Maria Nathalia Muñoz-Guerrero, Marien Palma-Parra, Carolina Becerra-Arias, Julián Alfredo Fernández-Niño

**Affiliations:** 1 Environmental and Labor Health Group, National Institute of Health, Bogotá 111321, Colombia; sdiaz@ins.gov.co (S.M.D.); rpalma@ins.gov.co (M.P.-P.); 2 Group Environmental Risk Factors, National Institute of Health, Bogotá 111321, Colombia; nmunozg@ins.gov.co; 3 Research Group on Health, Rehabilitation and Work (SARET), Manuela Beltrán University, Bucaramanga 680002, Colombia; carolina.becerra@umb.edu.co; 4 Department of Public Health, University of the North, Barranquilla 081007, Colombia

**Keywords:** mercury, mining, public health, exposure to environmental risks, Colombia

## Abstract

In Colombia, the inhabitants of the Mojana region have historically been subjected to high levels of environmental and occupational exposure to mercury; however, there are few robust data on the magnitude of this exposure and associated factors. This study aimed to describe the levels of mercury in the workers and inhabitants in this region, and to identify the main sociodemographic and occupational factors that are associated with this exposure. A cross-sectional study was conducted, in which mercury levels were determined in biological samples (blood, urine, hair) from 1119 people in the Mojana region. A questionnaire was also administered, which was adapted from the Global Mercury Assessment. Linear regression models were adjusted for the natural logarithm of mercury levels in blood, urine, and hair, using the factors that were explored as independent variables. The study reports high mercury levels in 35.0% of blood samples (95% CI 31.9–38.1%), 28.8% (95% CI 24.9–32.8%) of urine samples, and 56.3% (95% CI 53.1–59.5%) of hair samples. The reported source of water for consumption was associated with high levels of mercury (*p*-value < 0.05). We provide evidence of high levels of mercury exposure for the population in the Mojana region.

## 1. Introduction

One of the main contaminants that is associated with gold mining is mercury [1], which poses an imminent risk to human health, to the balance of the ecosystem, and to the sustainability of production processes, especially when in the form of methylmercury (MeHg). This is particularly the case in its gaseous form, which more easily spreads over vast distances and has a lifespan of up to 18 months [2,3]. MeHg is a powerful neurotoxin, and prolonged exposure to it increases the risk of neurological and cerebral damage, as well as cardiovascular diseases. Chronic exposure can even negatively affect immune and reproductive systems [4]. This is the most toxic form of Hg, since it easily bioaccumulates and biomagnifies up the food chain [5] until reaching levels that are harmful to humans [4]. While diverse sources of exposure to Hg exist, the primary ones are produced by anthropogenic emissions and the consumption of contaminated fish [6].

Small-scale artisanal mining (SSAM) is the main source of Hg emissions into the environment, and releases 1400 tons per year into water, air, and soil. In 2010, this source contributed 37% of the total anthropogenic emissions, and 24% in 2011 [6,7]. Mercury, which is used in alluvial mining, has become a recognized occupational and environmental risk factor [8] when this metal is not recovered after gold washing, or when it is burned and volatilized in vapors, or if it remains suspended in water [9]. Consequently, exposure to mercury currently poses a direct threat to human health. Previous reports have estimated that approximately 10 to 15 million people are involved in the extraction of gold through artisanal mining, mainly in countries in Africa, Asia, and South America. Additionally, according to calculations by the Global Mercury Assessment, around 3 million women and children worked in the artisanal mining sector in 2013 [6,10]. Colombia is probably the third source of mercury emissions, after China and Indonesia [11]. Gold mining is a type of this mining activity, which uses mercury amalgamation as the primary means of extracting gold, and can result in a loss of 1 to 2 g of mercury (released into the environment) per g of gold produced [7].

While workers are primarily exposed to mercury vapors released through occupational processes, the rest of the population is exposed to amalgam burning, given that most of the amalgamation processes occur very close to the miners’ homes. Therefore, mercury vapor might also affect workers’ families and other inhabitants [12]. Inhaled mercury vapor can easily pass through the walls of alveoli and enter the bloodstream. This can result in the body absorbing 80% of the inhaled amount, causing severe neurological, cardiovascular, or renal problems [13]. At amalgam burning sites, concentrations of mercury in the atmosphere have reached dangerously high values and usually exceed the limit for human exposure set by the World Health Organization (WHO) (1.0 μg/m^3^) [14,15].

The health effects that are produced by exposure to mercury vary depending on its chemical form (inorganic or organic) [16]. Exposure to mercury vapors during extractive work primarily affects the central nervous system and renal and thyroid functions [17]. Exposure to the organic form generates problems that mainly involve the neurodevelopment of the population living in areas surrounding the gold mines, which explains why the effects are more evident in children of mothers who are exposed to methylmercury in their diet [18,19,20,21,22,23,24].

La Mojana covers a wide geographic region in Colombia, and is bordered by the Cauca, San Jorge, and Magdalena rivers, which feed the piping and swamps in that region. This sub-region is crucial to the environmental regulation and ecological balance of Colombia’s coastal region. A key fauna resource is the water, which can be used for pisciculture, agriculture, and livestock. The northern portion of the zone (corresponding to flood areas) is the poorest in the region, where over 70% of families are poor. According to reports from 2005, the average annual income was U.S. $576 dollars for the entire region, and 85% of the population did not have its basic needs met, 57% of which lived in poverty conditions. Between 30% and 40% were illiterate [25]. La Mojana comprises four departments: Sucre, Antioquia, Bolívar, and Córdoba. It is one of the areas in Colombia with greater mercury contamination, especially from the widespread exploitation of gold, due to its hydrographic richness [26]. All of what has been mentioned makes the population in the zone a target for health problems that are related to mercury exposure. If identified early, before presenting the first symptoms, these can be controlled or even reversed for those with levels that have exceeded the defined limits.

Given the importance of occupational and environmental exposure to mercury in this region, as well as the exposure factors and the health effects mentioned above, the current study aimed to: (1) describe the demographic, environmental, and occupational conditions of people who have potentially been exposed to mercury in La Mojana, Colombia; (2) describe their mercury levels according to the type of exposure (environmental or occupational); (3) estimate the prevalence of the main symptoms of mercury exposure in the study population; and (4) evaluate associations among the main sociodemographic, occupational, and environmental factors that are related to exposure to mercury, as determined by the different biological matrices (blood, urine, and hair).

## 2. Materials and Methods

Background, design, and study population. A cross-sectional study was conducted between 2013 and 2015 with a population in 11 municipalities in La Mojana, Colombia. A total of 1119 people participated in the study. The study’s participants were distributed across the 11 municipalities as follows: Tiquisio 93, San Martin de Loba 102, Arenal 97, Guaranda 85, Majagual 57, San Marcos 95, Buriticá 114, Caucasia 114, El Bagre 111, Ayapel 163, and Montelíbano 88. These municipalities were selected because of the high number of workers in them that are dedicated to artisanal gold mining. Both workers (potential occupational exposure) and inhabitants (potential environmental exposure) were included as populations of interest.

Sample and selection criteria. Sample selection was based on a non-random sampling of each municipality. A total of 1119 participants agreed to voluntarily participate in the study, after awareness about the study was raised through meetings, parish messages, and local media, with the support of the Municipal Health Secretariats. The inclusion criteria for those potentially exposed to mercury through the environment were: (i) residing in one of these municipalities; (ii) being 18 years of age or older; and (iii) living in the area for at least six months. In terms of the group subject to occupational exposure to mercury, in addition to the above criteria, the selection included only people who had worked in mining in the region for at least six months. As exclusion criteria, the current study excluded individuals who reported having a neurological disease, or who had suffered a cerebrovascular event, or who suffered from a mental disorder, such as schizophrenia or bipolar disorder, according to self-reports.

Data collection. Participants were asked about their characteristics through a questionnaire that was adapted from the Global Mercury Assessment evaluation tool [10], which captures sociodemographic information and risk factors, such as: age; sex; schooling; social security; eating habits, with emphasis on the consumption of water and fish (frequency and type: herbivore or carnivore); and aspects related to mining work, including the amount of mercury used (ml or kg), frequency of exposure, years or months of exposure, and the use of retorts and personal protection elements. Also investigated was the presence, during the previous year, of signs and symptoms that were compatible with mercury poisoning and cigarette and alcohol consumption. This instrument was administered by professionals in bacteriology, microbiology, nursing, and epidemiology, who received previous training in the standardized evaluation of the participants.

Quantification of mercury. Samples taken of each participant included venous blood (10 mL), urine (50 mL), and hair from the occipital region of the scalp (10 mg). Blood was collected with a tube using EDTA (Ethylenediaminetetraacetic acid) as an anticoagulant, and the urine was collected in polypropylene bottles with a screw cap. These two samples were kept in refrigeration until the analysis was carried out. The hair was kept in polyethylene bags at room temperature. The identification of each sample was masked with a barcode for both the interviewers and the laboratory analysts, so that only the field coordinator possessed a master list with the names of the participants. The samples were analyzed with DMA-80 TriCell Milestone (Milestone INC, Shelton, U.S) equipment according to EPA (Environmental Protection Agency) method 7473 (thermal decomposition, amalgamation, and atomic absorption spectroscopy) [27] in the Environmental and Labor-Health laboratory of the National Institute of Health (NIH). Cold vapor atomic absorption spectrometry (CVAAS, EPA, Cincinnati, U.S) was used to process the blood samples. The method used to determine mercury in the different biological matrices was standardized by the NIH, based on international patterns for each matrix, with the quality control system that was established by this entity. The limit of detection for the method was 0.87 μg/L, and the limit of quantification was 2.6 μg/L. The values below the limit of detection (only 3% for the blood samples) were imputed to a value of 0.44 (half between 0 and 0.87). With this imputation the medians are not affected, and the estimators that are obtained from the regressions are practically the same.

Operationalization of mercury exposure. The definition established by the National Institute of Health was used for a person with mercury poisoning. This corresponded to a “resident in the area with a history of exposure to mercury or a high frequency of consumption of local fish, who presents one or more symptoms, evaluated during the previous year (including tremors, metallic taste, memory alterations, mood alterations, such as depression, as well as insomnia, excessive salivation, and headache), and associated with the presence of mercury levels, detected in the laboratory, that exceed permissible levels in one of the biological samples taken (>7 μg/L in urine, or >5 μg/L in whole blood, or >1 μg/g of hair)” [28]. However, blood values over 5 μg/L, urine over 7 μg/L, and hair over 1 μg/g were considered indicators of toxicity for those with potential environmental exposure, while values higher than 15 μg/L in blood, 25 μg/L in urine, and 2 μg/g in hair were considered indicators of toxicity for potential occupational exposure [29].

Statistical analysis. The qualitative variables were summarized as proportions with their respective 95% confidence intervals. Quantitative variables were described using measures of central tendency (mean and median) and dispersion (standard deviation (±) and interquartile range (IQR)). In the exploratory analyses, the quantitative variables were described with histograms and p-norm and q-norm graphs. Differences between groups were identified with the Student’s t-test in the case of symmetric continuous variables, and the non-parametric Mann–Whitney test was used for non-symmetric variables. The chi-square test was used for qualitative variables. For the bivariate analyses, each independent variable was explored with respect to the mercury level in its continuous and dichotomous forms. For the multiple components, the variables that were reported in the literature as being associated with this level were used for the statistical models of each sample. Given that the mercury levels in blood, urine, and hair did not follow a normal distribution, a linear regression model was adjusted for the natural logarithm of the levels in each matrix, with sociodemographic, environmental, and occupational factors as independent variables. The coefficients that were obtained in each model were exponentiated in order to be interpreted as percentage changes in the expected level of the response variable.

All of the assumptions of the regression models were verified with the use of graphical and numerical methods. The distribution of generalized residues for the assumptions of linear regression models was explored, including normality, independence, homoscedasticity, and collinearity. As part of the diagnostic of the final adjusted model, the value of R² was also taken into account. The influence of extreme values was explored and discarded based on the prediction and graph of leverage, and the analysis of potential influential or extreme values. All associations with a p-value less than 0.05 were considered statistically significant. The analyses were performed with STATA 12 (StataCorp College Station, TX, USA).

Ethical considerations. For the execution of the present study, informed consent was obtained from each participant who met the selection criteria before the collection of information and samples. Confidentiality in the handling of information was safeguarded at all times, and the basic principles pertaining to research on human beings were followed (justice, beneficence, and non-maleficence). This research took into account the ethical considerations raised by the Colombian Ministry of Health in Resolution 8430 of 1993, and the Declaration of Helsinki. Additionally, this research was approved by the Research Ethics Committee of the University of the Andes, Colombia (approval number 459/2015).

## 3. Results

Sociodemographic and occupational characteristics of the participants. This work studied 487 (43.5%) people who were potentially exposed to mercury at work and 632 (56.5%) who were potentially exposed to mercury in the environment. The average age was 40.5 ± 14.1 years; 66.5% of the records corresponded to men. A total of 7.9% of participants were illiterate, while 71.7% had some formal education (complete or incomplete elementary or secondary school). The remaining 20.4% had higher education (complete or incomplete). The median mercury level was 5.5 μg/L in blood (interquartile range (IQR): 2.7–11.2 μg/L), 8.15 μg/L in urine (IQR 4.6–17.5 μg/L), and 1.5 μg/L in hair (IQR 0.8–3.1 μg/L). Regarding the time during which people lived in the region, 24.7% had lived there for 6 or fewer years, 26.7% had lived there from 6 to 22 years, 16.3% from 22 to 30 years, and 32.4% for more than 30 years.

With respect to the time of exposure to mercury from mining activities, a total of 59.6% reported being over 30 years old, and, therefore, they were subjected to chronic exposure. In addition, 19.3% had worked in mining activities for less than 6 years, 8.7% between 6 and <14 years, 6.4% between 14 and <22 years, and 5.9% between 22 and 30 years. The median value for this variable was 72 months (IQR 36–234), with a minimum value of 6 and a maximum of 600 months. Moreover, regarding occupational exposure, 38.5% reported the use of mercury in the workplace, of which 30.5% still used it at the time of the survey. Of those exposed to mercury due to their occupation, 17.5% was exposed weekly, 9.3% daily, and 8.2% monthly. A total of 55.9% had contact with 50–1000 g of mercury per day, and 24.8% were in contact with 1000 and 10,000 g per day. A total of 11.6% had the least contact (50 g or less) and 7.6% the highest (>10,000 and ≤250,000 g). Overall, we report a median of 875 g/day (IQR 250–2500) for the occupational use of mercury, with a minimum value of 1 and a maximum value of 250,000 g/day.

Mercury levels in the participants. A total of 35.0% (95% CI 31.9–38.1%) of the blood samples presented mercury levels that were higher than the cutoff points for each type of exposure (occupational >15 μg/L and environmental >5 μg/L). In the case of urine, 28.8% (CI 95% 24.9–32.8%) of the participants showed levels above the limit (25 μg/L for occupational exposure and 7 μg/L for environmental exposure). Moreover, 56.3% (CI 95% 53.1–59.5%) of the people presented altered values of mercury concentration in their hair (a limit of 2 μg/g for occupational exposure and 1 μg/g for environmental exposure). In general, 56.7% (CI 95% 53.8–59.6) of the respondents showed increases in mercury levels in some of the studied samples. In the case of men, 53.8% (95% CI 50.2–57.4) of the total presented alteration in levels, compared to 62.7% (95% CI 57.8–67.6) of women.

Correlation among the analyzed biological matrices. The natural logarithms of the three matrices were analyzed using Spearman correlation to identify the relationships among them, based on the rho correlation coefficient and the p-value, as shown in Table 1. The mercury levels in blood showed a positive correlation with urine and hair levels, and urine levels were correlated with hair levels. Nevertheless, the highest correlation found was between blood and hair levels, as indicated by the coefficient value presented in Table 1.

Bivariate analysis of exposure to mercury. We report that men presented a higher concentration of mercury in blood than women, with a statistically significant difference (*p* < 0.05). Similar results were also found for levels in hair and urine. The majority of surveyed men reported working with mercury (42.6%), whereas only 6.4% of women responded affirmatively to this question. Thus, occupational exposure was closely related to sex, and environmental exposure, especially according to place of residence, frequently occurred in women. Table 2 shows the main characteristics of the participants according to type of exposure to mercury and sex. It is notable that the differences in the mercury levels (in all three matrices) of men and women were not statistically significant among the group of those who were potentially exposed at work, whereas significantly statistical differences in mercury levels (in blood and hair) were found between men and women who were potentially subjected to environmental exposure, which were higher for men in both cases (Table 2). Regarding the prevalence of high levels (based on the previously defined cut-off points for each biological matrix), these were higher in women than in men with the same exposure, although they were not statistically significant. However, when evaluating symptoms, in terms of occupational exposure, we found that men presented a higher frequency of insomnia, tremors, and headache (*p* < 0.00). In terms of environmental exposure, alterations in memory were higher in women, while tremors, insomnia, headache, and depression were statistically higher in men.

With regard to associations between the studied factors and exposure to mercury based on the biological matrices, the simple linear regression models indicated the following as being related to higher mercury levels: the improper use or lack of personal protection elements, the use of mercury at work and current use, storage of amalgam at home, alcohol and cigarette consumption, and the source of drinking water (Table 3). According to the multivariable regression for levels in blood, urine, and hair, in the continuous form, the water source was the only variable associated with the outcome, for all the samples, especially bottles and rivers or ravines (Table 4).

## 4. Discussion

In Colombia, as in the world, mining activity takes place informally, with few conditions to guarantee the workers’ health and safety. In La Mojana, these informal workers usually present alterations in health (acute or chronic poisoning) that are not diagnosed due to a lack of employee health monitoring, as in other regions of Colombia [30].

Roughly 33% of the study’s participants reported as having lived in the area for more than 30 years, which indicates chronic exposure since these municipalities are located within the area of mining influence. We report high use of mercury in both quantity and frequency, although the median blood levels remained below the limit for occupational exposure (15 μg/L). However, in the case of environmental exposure, we found that a total of 72.3% (CI 95% 68.6–76.1) exceeded the maximum value (5 μg/L). Additionally, the differences found in the mercury levels of men and women suggest different types of exposure. Thus, for those who were subjected to environmental exposure, women presented lower levels of mercury in blood and hair. This finding indicates that the differences found between men and women can mostly be explained by the work, and, therefore, they tend to disappear when comparing men and women who work. Meanwhile, differences were found when comparing levels in men who are potentially exposed to mercury at work with men who are potentially exposed environmentally, or when comparing women who are potentially exposed at work with women who are potentially exposed environmentally. This demonstrates the importance of occupational exposure for both sexes, rather than the role of sex per se, which is more closely linked with work-related gender roles. In this sense, the difference that remains between men and women who were potentially exposed environmentally can be explained by the possibility of some of the men being former workers or workers who did not report their occupation.

Regarding the factors that were associated with high levels of mercury in any of the three samples, after adjusting for potential confounders, only the source of water for consumption remained associated with the response variable in the three regression models. In terms of this variable, it is worth noting that a statistically significant association was found for the categories corresponding to consumption of bottled and river or ravine water. Although unexpected, this finding suggests that the bottling of water in that locality may not follow regular procedures, which would contribute to the contamination of a product sold in the area and consumed by those who are exposed at the workplace and environmentally [31]. In turn, the river and ravine water that is bottled without adequate treatment processes would also result in the association found with this water source category. However, this requires testing and substantiating. Other possible sources to consider include ingestion of soil, inhalation of dust, or skin contact with mercury [32]. In its inorganic form, mercury can be found in fossil fuel emissions, dental amalgams, and commercial products, such as skin lotion, germicidal soap, medications, whitening powders, fluorescent bulbs, and batteries, among others [33]. It is, therefore, important to consider these elements when evaluating factors that are associated with elevated mercury levels, though it was not possible to take them into account in the present study.

This finding is also consistent with the results of the high correlation found between the mercury levels of the blood samples and those measured in hair (Table 1), which could be explained due to the coastal location and the mining activity of the region, and would be a manifestation of the exposure having a cumulative effect [34], but also of a recent persistent exposure. Additionally, hair levels are an alternative for the measurement of organic Hg levels, since they reflect the chronic exposure to mercury in a sample of great stability and without invasiveness. However, inter-individual variability and sample contamination should be also considered [35]. In this way, the high correlations found in the values in different samples would make it clear that, in the studied population, exposure to mercury through water has an almost permanent effect, with an association between levels in hair and blood, which would manifest in long and short-term exposures, respectively. Previous studies have found similar correlations [35,36].

The symptoms found in the population showed statistical differences between men and women, which is likely the result of the occupation being closely linked to men’s work, rather than environmental exposure. Thus, for occupational exposure, the differences in mercury levels in the biological matrices of men and women disappeared, while for environmental exposure they remained significantly higher for men, at least for the blood and hair values. This result indicates that men’s history of occupational exposure may be different, even when they do not currently work in mining, or because they perform another activity that poses a greater risk than that of women who traditionally spend most of their time at home. In addition, one possible argument for the differences between sexes could be the existence of underlying mechanisms, which need to be fully elucidated. These include interaction with sulfhydryl groups, destabilization of microtubules, perturbation of intracellular calcium, and the formation of reactive species of oxygen, which tend to have a more determinant role in the neurotoxicity of mercury for men than for women [37]. However, we also want to note that, although few women work in mining, when they have performed this work their average levels of mercury have reached similar values as those observed in men.

Although occupational exposure to mercury has been documented as a factor responsible for adverse effects on human health, environmental levels at high doses can also present effects, which is known as indirect exposure. This exposure becomes more relevant when taking into account that some individual factors are capable of modifying the personal response to contact with mercury, through genetic determinants. These, in turn, could contribute to inter-individual variations in the levels of the biological markers, such as urine, hair, and blood, as well as on health effects, especially neurotoxicity. This may be due to certain genetic mutations that can affect the retention of methylmercury in the body, specifically, the metabolism of glutathione and metallothioneins. However, the mechanisms involved in these relationships remain mostly unknown [38,39]. Hence, the importance of considering both environmental and occupational factors, and even personal factors (such as genetic traits) that could determine how exposure to mercury impacts an individuals’ health.

In terms of limitations, it is worth noting that the symptoms that were evaluated were identified according to self-reports by the participants. Thus, given the non-specific nature of the symptoms of mercury poisoning, the fact that this evaluation was not based on a medical history could have resulted in over-reporting, that is, not the result of exposure to mercury but rather to other conditions, such as cigarette smoking, alcohol, and exposure to dust and other substances that are present in mines [38]. Therefore, the identification of signs and symptoms should include a medical evaluation and the use of standardized instruments to reduce subjectivity at the time of reporting and diagnosing mercury poisoning. Consequently, the prevalence of the symptoms studied herein could be explained by other exposures that produce similar effects, in addition to exposure to mercury.

Another limitation corresponds to the absence of a control group, which was due to the lack of a population group without exposure, or given that the Colombian population is characterized by a lower mercury level. Therefore, the entire study population had some level of occupational or environmental exposure because they lived in areas close to mining activity. Additionally, considering the type of design used, it was possible to generate hypotheses and evaluations at a given moment in time, but it was not possible to determine, with high accuracy, that exposure to mercury preceded the symptoms, especially since this was a cross-sectional study. However, given the evaluation of chronic exposure to mercury based on levels in hair, whose prevalence was 56.3%, it is reasonable to assume that exposure to mercury preceded symptoms, despite this study being a cross-sectional one.

In terms of the potential biases that may have occurred, selection bias cannot be ruled out, since the study was carried out in an area of exposure where high levels of mercury were more likely to be found in biological samples of this population than in the general population. Despite the above limitations, this is the first study in Colombia to evaluate the mining population in order to identify factors associated with high mercury levels. Regarding information bias, it was not possible to avoid this given the self-reporting of symptoms. Moreover, investigation of the current occupation of the participants could have contributed to reference bias towards the healthy worker. In this case, those who worked in mining at the time of the survey may have been those with a better health status, whereas those with some type of pathological condition may have been inactive at the time of the current survey.

To reduce the influence of this information bias, our study was carried out throughout the entire area, that is, on a population basis, in the 11 municipalities. We also managed confusion bias when performing the statistical analyses, for which the multiple regressions took into account the potentially confounding variables reported in the literature. However, the multiple regression model did not rule out confounding due to unmeasured or unknown variables.

It is worth mentioning the advantages of the present findings. First, the major goal proposed herein was to evaluate factors that are associated with exposure to mercury. This should also take into account the need to include a baseline population in order to establish a precedent for the health status of the study population, based not on assumptions but rather on scientific findings. Also worth noting is that previous studies evaluated populations with distinct environmental circumstances (i.e., climate, humidity, and temperature, among others) and social conditions [40,41]. Additionally, since most of the previous reports were descriptive studies, our study would be one of the first to use analytical approaches.

Another advantage was the use of standardized techniques, by the NIH, to determine mercury in biological samples. That contributed to diminishing potential information bias related to systematic errors, which could occur when using non-parametrized techniques. Moreover, the collection, packaging, and processing of the samples, as well as data collection and processing (carried out in NIH facilities), were conducted by appropriately trained personnel with sufficient experience in each case, which contributes to the validity of the present results.

## 5. Conclusions

In conclusion, our findings demonstrate high levels of mercury in the population in the Mojana region of Colombia (residents and others working in mining). Additionally, it is one of the first analytical studies in the country to identify factors associated with high mercury levels in three samples taken from a population in La Mojana, although only the source of water for consumption was finally identified as an associated factor. The findings presented herein can contribute to public health by identifying the conditions that directly and indirectly influence mercury levels in the exposed population, so that, in the medium term, mining conditions can be improved and evidence provided to reinforce the use of safer alternatives when conducting this mining activity.

## Figures and Tables

**Table 1:** The Spearman correlation for the natural logarithm of mercury levels in blood, urine, and hair.

Matrix	Mercury in Blood	Mercury in Urine	Mercury in Hair
Mercury in blood	1 *		
Mercury in urine	0.326 * 461 ^ <0.001 ^ǂ^	1 *	
Mercury in Hair	0.763 * 766 ^ <0.001 ^ǂ^	0.243 * 401 ^ <0.001 ^ǂ^	1 *

* Rho: Spearman correlation coefficient; ^ number of observations; ^ǂ^ *p*-value.

**Table 2:** Characteristics of the participants according to type of exposure and sex in the La Mojana region, 2015.

Variable	Occupational Exposure (n = 487)				*p*-Value	Environmental Exposure (n = 632)				*p*-Value
	Men n = 451		Women n = 36			Men n = 293		Women n = 339		
	$\bar{X}$ o %	CI 95%	$\bar{X}$ o %	CI 95%		$\bar{X}$ o %	CI 95%	$\bar{X}$ o %	CI 95%	
Biological matrix										
Mercury in blood μg/L *	7.4	3.6–14.8	7.6	4.2–15.3	0.75	5.1	2.6–10	3.6	1.9–7.8	<0.00
Mercury in urine μg/L *	9.9	5.1–24	14	5.2–26.5	0.46	6.1	3.6–9.1	5.6	4–9.5	0.81
Mercury in hair μg/g *	2.1	1.0–4.2	2.3	1.2–3.2	0.75	1.4	0.8–2.9	1.2	0.6–2.1	<0.00
Education					<0.00					0.05
Elementary	55.7	51.1–60.3	38.9	22.7–55.1		45.1	39.3–50.8	37.5	32.3–42.6	
Secondary	38.6	34.1–43.1	38.9	22.7–55.1		26.3	21.2–31.3	30.1	25.2–34.9	
Technical/Undergraduate degree	5.8	3.6–7.9	22.2	8.4–36.0		27.6	22.5–32.8	32.4	27.4–37.5	
Postgraduate	No observations		No observations			1.0	0.1–2.1	No observations		
Use of mercury										
Length of use (months) *	72	36–240	54	24–144	0.29	No observations				
Quantity (g/day) *	1000	400–2500	500	50–500	<0.00					
Frequency of the use of Hg					0.45					
Daily	26.8	21.9–31.7	20.8	4.2–37.5		No observations				
Weekly	49.2	43.7–54.7	62.5	42.6–82.4						
Monthly	23.9	19.3–28.7	16.7	1.4–31.9						
Consumption habits										
Current cigarette consumption	19.1	15.4–22.7	8.3	0.1–17.5	0.18	9.9	6.5–13.3	5.6	3.1–8.1	<0.00
Current alcohol consumption	79.2	75.4–82.9	52.8	36.2–69.4	<0.00	65.9	60.4–71.3	44.8	39.5–50.2	<0.00
Drinking water source					0.25					0.02
Faucet	34.4	29.9–38.8	41.7	25.3–58.1		46.8	41.0–52.5	42.5	37.2–47.8	
Bottle	7.8	5.3–10.2	13.9	2.4–25.4		10.2	6.8–13.7	7.1	4.3–9.8	
River/stream	18.8	15.2–22.5	11.1	0.7–21.6		19.8	15.2–24.4	20.6	16.3–24.9	
Well water	36.8	32.3–41.3	27.8	12.9–42.7		19.5	14.9–24.0	28.6	26.8–33.4	
Others	2.2	0.8–3.6	5.6	2.1–13.2		3.7	1.6–5.9	1.2	0.0–2.3	
Fish consumption frequency					0.61					0.18
Never	3.5	1.8–5.3	2.8	2.6–8.2		4.8	2.3–7.2	5.0	2.7–7.3	
Once per month	32.2	27.8–36.5	25.0	10.6–39.4		31.1	25.7–36.4	32.7	27.7–37.8	
Once per week	28.4	24.2–32.6	25.0	10.6–39.4		22.2	17.4–26.9	28.9	24.1–33.8	
2–4 times per week	26.4	22.3–30.5	38.9	22.7–55.1		32.4	27.0–37.8	24.8	20.2–29.4	
Daily	9.5	6.8–12.2	8.3	0.9–17.5		9.6	6.2–12.9	8.6	5.6–11.5	
Prevalence of high values of mercury (in any matrix)	48.3	43.7–52.9	61.1	44.9–77.3	0.14	62.1	56.5–67.7	62.8	57.7–68.0	0.85
Symptom										
Metallic flavor	12.9	9.8–15.9	19.4	6.3–32.6	0.26	14.7	10.6–18.7	16.8	12.8–20.8	0.46
Salivation	74.7	70.7–78.8	72.2	57.3–87.1	0.74	76.5	71.6–81.3	78.5	74.1–82.9	0.54
Memory disorders	40.1	35.6–44.7	38.9	22.7–55.1	0.95	33.1	27.7–38.5	46.1	40.1–51.6	<0.00
Tremor	76.1	72.1–80.0	55.6	39.9–72.1	<0.00	73.0	67.9–78.1	65.2	60.1–70.3	0.03
Insomnia	61.9	57.4–66.4	38.9	22.7–55.1	<0.00	60.1	54.4–65.7	48.9	43.6–54.3	<0.00
Headache	68.5	64.2–78.8	44.4	27.9–60.9	<0.00	72.3	67.2–77.5	46.9	41.6–52.2	<0.00
Depression	68.7	64.4–73.0	58.3	41.9–74.7	0.20	70.3	65.1–75.6	60.2	59.4–65.4	<0.00

The proportion is presented for qualitative variables and the average for quantitative variables. In some cases, the (*) median and interquartile range (IQR) are presented due to the asymmetric distribution of the variable.

**Table 3:** The results of the bivariate analysis between independent variables and mercury levels in biological matrices in the La Mojana region, 2015.

Model	β	exp^β^	CI 95%	*p*-Value
Mercury in Blood (nl of µg/L)				
Alcohol consumption	0.2	0.9	0.7–0.9	0.04
Source of drinking water (Faucet)	Reference			
Bottle	0.6	1.7	1.4–2.2	<0.01
Well water	0.4	1.5	1.3–1.7	<0.01
Use of PPE	−0.0	0.9	0.9–0.9	<0.01
Technical/Undergraduate degree	−0.4	1.5	0.6–0.8	<0.01
Storage of amalgam at home	−0.0	0.9	0.9–0.9	<0.01
Tobacco consumption	−0.0	0.9	0.9–0.9	<0.01
Use of mercury at work	−0.0	0.9	0.9–0.9	<0.01
Mercury in urine (nl of μg/L)				
Source of drinking water (faucet)	Reference			
Bottle	1.1	3.1	2.2–4.4	<0.01
River/ravine	−0.5	0.6	0.4–0.8	<0.01
Use of PPE	−0.0	0.9	0.9–0.9	<0.01
Technical/ Undergraduate degree	0.5	1.6	1.3–2.0	<0.01
Storage of amalgam at home	−0.0	0.9	0.9–0.9	<0.01
Use of mercury at work	−0.0	0.9	0.9–0.9	<0.01
Mercury in hair (nl of μg/g)				
Source of drinking water (faucet)	Reference			
Bottle	0.5	1.7	1.3–2.1	<0.01
Well water	0.4	1.5	1.3–1.8	<0.01
Use of PPE	−0.0	0.9	0.9–0.9	<0.01
Technical/Graduate	−0.5	0.6	0.5–0.7	<0.01
Storage of amalgam at home	−0.0	0.9	0.9–0.9	<0.01
Consumption of tobacco	−0.0	0.9	0.9–0.9	<0.01
Use of mercury at work	−0.0	0.9	0.9–0.9	<0.01

PEE: personal protection elements. nl = natural logarithm. exp^β^ = The coefficients were exponentiated to be interpreted as average percentage changes in the response variable at the original scale.

**Table 4:** The multiple linear regression models for mercury levels in different biological matrices in the La Mojana region, 2015.

Model	β	exp^β^	CI 95%	*p*-Value
Mercury in Blood (nl of µg/L)				
Age	0.01	1.0	0.9–1.0	0.21
Sex (men)	Reference			
Women	−0.16	0.85	0.5–1.4	0.55
Source of drinking water (faucet)	Reference			
Bottle	0.88	2.42	1.4–4.1	<0.01
River/ravine	−0.38	0.68	0.5–1.0	0.05
Well water	0.16	1.27	0.9–1.6	0.27
Others	0.07	1.18	0.4–3.2	0.89
Education (elementary)	Reference			
Secondary	0.05	1.05	0.8–1.4	0.70
Technical/Undergraduate degree	0.19	1.21	0.7–2.2	0.54
Mining length (months)	−0.00	1.0	1.0–1.0	0.47
Use of mercury at work	0.57	1.77	0.4–8.2	0.46
Fish consumption frequency (never)	Reference			
Once per month	0.08	1.1	0.5–2.3	0.83
Once per week	0.16	1.17	0.5–2.5	0.68
2–4 times/week	−0.17	0.84	0.4–1.8	0.65
Daily	0.24	1.27	0.5–3.0	0.58
Mercury in urine (nl of μg/L)				
Age	0.00	1.00	0.9–1.0	0.27
Sex (Men)	Reference			
Women	0.04	1.04	0.6–1.9	0.91
Source of drinking water (faucet)	Reference			
Bottle	1.36	3.89	2.1–7.1	<0.01
River/ravine	−0.65	0.52	0.3–0.8	0.01
Well water	−0.09	0.91	0.6–1.3	0.57
Others	−0.25	0.78	0.2–7.0	0.78
Education (elementary)	Reference			
Secondary	0.25	1.28	0.9–1.8	0.12
Technical/ Undergraduate degree	0.62	1.86	0.9–3.6	0.06
Mining length (months)	−0.00	1.00	1.0–1.0	0.28
Use of mercury at work	0.09	1.09	0.1–12.3	0.94
Fish consumption frequency (never)	Reference			
Once per month	0.08	1.08	0.5–2.5	0.85
Once per week	−0.24	0.79	0.3–1.8	0.57
2 –4 times/week	−0.23	0.79	0.3–1.9	0.59
Daily	−0.02	0.98	0.4–2.6	0.97
Mercury in hair (nl of μg/g)				
Age	0.01	1.0	0.9–1.0	0.14
Sex (Men)	Reference			
Women	0.07	1.1	0.7–1.7	0.75
Source of drinking water (faucet)	Reference			
Bottle	0.55	1.6	1.0–2.9	0.04
River/ravine	−0.47	0.6	0.4–0.9	0.01
Well water	0.17	1.2	0.9–1.6	0.24
Others	−0.06	0.9	0.2–3.7	0.94
Education (elementary)	Reference			
Secondary	0.12	1.1	0.9–1.5	0.38
Technical/ Undergraduate degree	−0.07	0.9	0.5–1.6	0.78
Mining length (months)	−0.00	1.0	1.0–1.0	0.44
Use of mercury at work	Not estimable			
Fish consumption frequency (never)	Reference			
Once per month	−0.2	0.8	0.5–1.6	0.63
Once per week	−0.05	0.9	0.5–1.8	0.88
2–4 times/week	−0.3	0.7	0.4–1.4	0.32
Daily	−0.03	0.9	0.5–1.9	0.92

nl = natural logarithm. exp^β^ = The coefficients were exponentiated to be interpreted as average percentage changes in the response variable at the original scale.
